# Supplementary material for: Toy model of harmonic and sum frequency generation in 2D dielectric nanostructures
Source: Sci Rep. 2021 Oct 11;11:20120. doi: 10.1038/s41598-021-99567-4 (PMC8505426; doi:10.1038/s41598-021-99567-4)
Supplement: Supplementary file 1 — Supplementary Information. [file 41598_2021_99567_MOESM1_ESM.docx]

Supplementary Information

**Toy model of harmonic and sum frequency generation in 2D dielectric nanostructures**

**JIE XU^1^, VASSILI SAVINOV^1^, AND ERIC PLUM^1,*^**

1 Optoelectronics Research Centre and Centre for Photonic Metamaterials, University of Southampton, Southampton, SO17 1BJ, United Kingdom

*erp@orc.soton.ac.uk

**Fig. S1.** Sum frequency generation in a nanoparticle of D4 symmetry. Frequency dependence of the electric dipole moment of a square particle consisting of 25 atoms in response to optical pumping at a combination of two frequencies, *ω*_p_ and 0.2*ω*_p_. *P_y,x_* indicates the *y*-component of the particle’s electric dipole moment caused by *x*-polarized pumping. For either *x*- or *y*-polarized pumping, the orientations of pump polarization and generated dipole are parallel, and the generated dipoles have the same magnitude for both cases.

**Fig. S2.** Magnitude (colours) and sign (“+” and “-”) of the dipole moment per atom, ***d***, generated at lowest-order sum frequencies in response to pumping at frequencies *ω*_p_ and 0.1*ω*_p_. The top (bottom) row shows the dipole component orthogonal (parallel) to the pump polarization. Stacked images for different – either even or odd – sum frequencies show the same qualitative behaviour.

**Fig. S3.** Scaling of the triangular particle’s electric dipole moment at harmonic frequencies with the pump field amplitude for the cases of Fig. 3.

**Fig. S4.** Sum frequency generation in a structure of D3 symmetry with co-polarized pumping. (a)-(c) Frequency dependence of the electric dipole moment of the triangular particle of 28 atoms (inset) in response to pumping at a combination of two frequencies, *ω*_p_ and 0.1*ω*_p_ for different pump electric field amplitudes of (a) *E_0_*, (b) E_0_/2, (c)E_0_/4.

**Fig. S5.** Sum frequency generation in a structure of D3 symmetry with cross-polarized pumping. (a)-(c) Frequency dependence of the electric dipole moment of the triangular particle (inset) in response to pumping at two frequencies, *ω*_p_ and 0.1*ω*_p_, where the pump fields at different frequencies have orthogonal polarizations. $P_{i,jk}$ refers to the *i*-component of the dipole moment caused by *j*-polarized pump field at *ω*_p_ and *k*-polarized pump field at 0.1*ω*_p_, where *i,j,k* is *x* or *y*. Different panels show different pump electric field amplitudes of (a) *E_0_*, (b) E_0_/2, (c)E_0_/4.
